# Left Internal Mammary Artery Transection: A Rare Complication of Pericardiocentesis

**DOI:** 10.7759/cureus.6677

**Published:** 2020-01-16

**Authors:** Raunak Nair, Oscar Perez, Bassel Akbik, Emad Nukta

**Affiliations:** 1 Internal Medicine, Cleveland Clinic - Fairview Hospital, Cleveland, USA; 2 Critical Care Medicine, Cleveland Clinic, Cleveland, USA; 3 Cardiovascular Medicine, Cleveland Clinic, Cleveland, USA

**Keywords:** pericardiocentesis, pericardial tamponade, lima rupture, subxiphoid approach, echocardiogram, fluoroscopy

## Abstract

Pericardiocentesis is a procedure performed to aspirate fluid from the pericardial space. It is most often performed as an acute therapeutic procedure in cases of massive pericardial effusion or pericardial tamponade. Our case describes an unusual complication of pericardiocentesis leading to hemorrhagic pericardial effusion and hemodynamic compromise.

## Introduction

Pericardiocentesis or ‘pericardial tap’ is an invasive procedure done to remove fluid from the pericardial sac. It is a lifesaving intervention that is performed in cases when hemodynamic compromise occurs due to a large pericardial effusion or cardiac tamponade. It is usually performed percutaneously under echocardiogram (echo) or fluoroscopy guidance via one of three approaches: (1) subxiphoid, (2) parasternal, or (3) apical approach [1]. Though several complications can be seen following pericardiocentesis, transection of the internal mammary artery via a subxiphoid approach has not been described [2,3]. We present a rare case of hemorrhagic pericardial effusion causing tamponade due to transection of the left internal mammary artery (LIMA) following fluoroscopy-guided percutaneous pericardiocentesis.

## Case presentation

**Figure 1:**
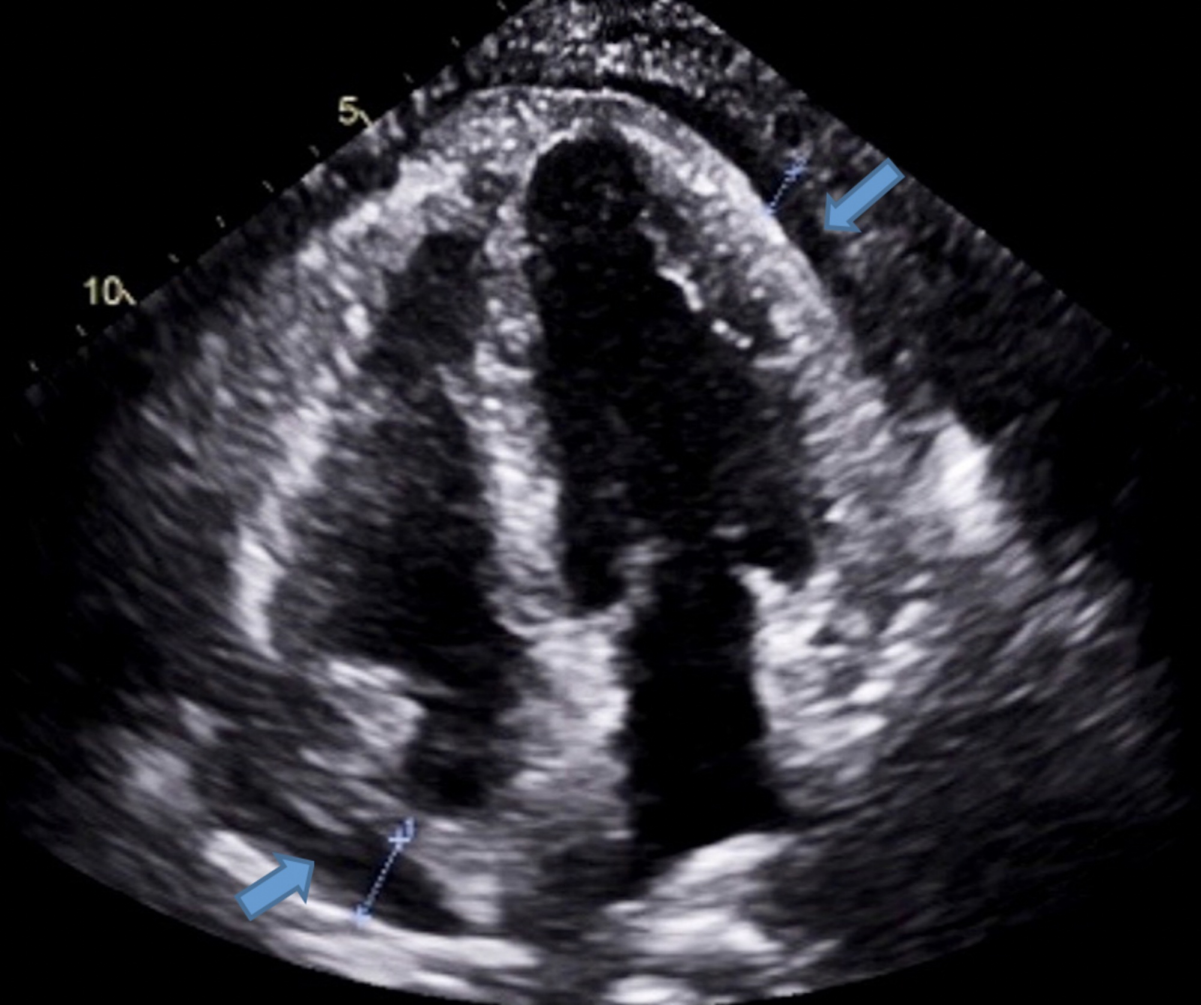
Apical 4 chamber view showing a moderate pericardial effusion.

A 65-year-old lady with a past medical history significant for chronic obstructive pulmonary disease (COPD), hypertension, and hypothyroidism presented to our hospital for evaluation of dizziness. The patient was recently discharged from our hospital after being treated for COPD exacerbation. A computed tomography (CT) scan of her chest done at that time had revealed new-onset mediastinal lymphadenopathy and the presence of a moderate pericardial effusion (Figure 1). She had undergone bronchoscopy and biopsy of the mediastinal mass and was being followed by pulmonology and cardiology as an outpatient.

**Figure 2:**
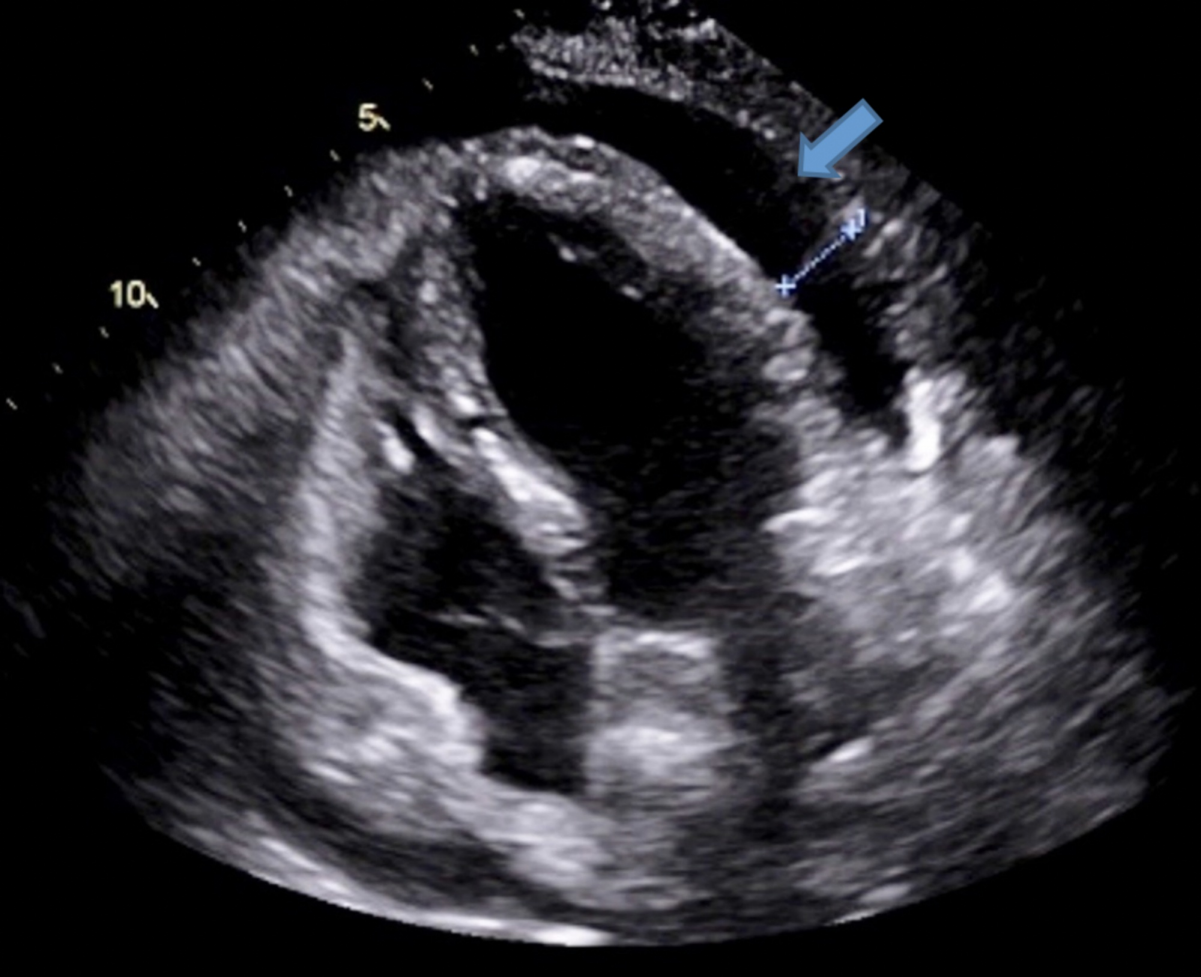
Apical 4 chamber view showing an interval increase in the size of the effusion.

Upon presentation this time, the patient complained of dizziness for a week, which was worse on standing. She also described multiple near-fainting spells. She denied other neurological symptoms. She also denied chest pain, dyspnea, palpitations, or tinnitus. Her vital signs on presentation were within normal limits, and her examination did not reveal any positive findings. Initial lab tests including blood counts, metabolic profile, thyroid-stimulating hormone, cardiac enzymes, arterial blood gas, and electrocardiogram were unremarkable. Imaging tests including chest X-ray and CT scan of the head also did not reveal any acute abnormalities. Her orthostatic vitals were significantly positive. Given her recent diagnosis of pericardial effusion and history suggestive of presyncope, a limited echo was ordered to assess any increase in the size of the effusion. The repeat echo revealed that the effusion had significantly increased in size (Figure 2), along with findings suggestive of pre-tamponade physiology.

**Figure 3:**
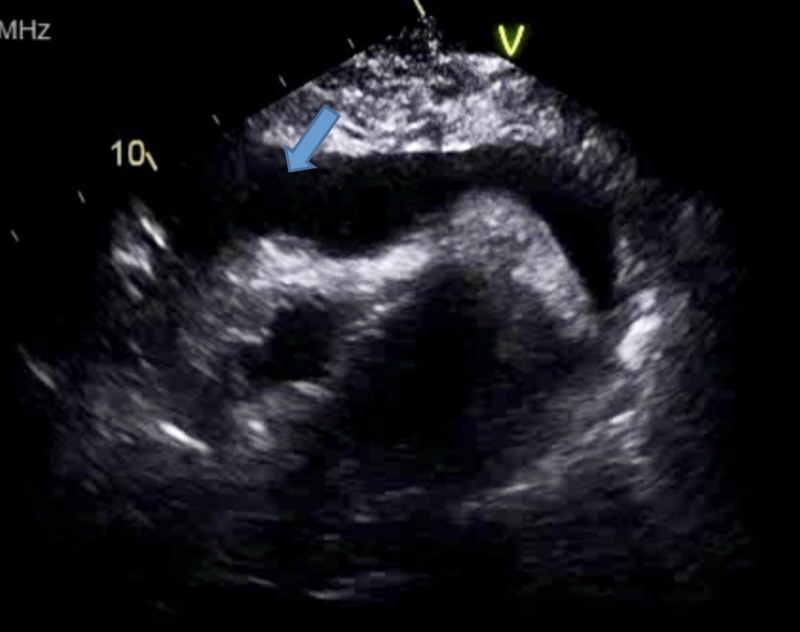
Apical view showing a large pericardial effusion.

Interventional cardiology was consulted, and the patient was taken to the cardiac catheterization lab for immediate pericardiocentesis under fluoroscopy guidance. A subxiphoid approach was used, and approximately 600 mL of bloody fluid was drained from the pericardial space. A catheter was also left in place to drain overnight. Overnight, the patient started becoming progressively short of breath and hypoxic. She was given breathing treatments and placed on 6 L supplemental oxygen via a nasal cannula. She subsequently became hypotensive, pale, and tachycardic, and there was a sudden gush of around 200 cc blood in the pericardial drain. The patient was given IV fluid boluses and started on norepinephrine infusion. Around 1.5 L of hemorrhagic fluid was drained via the pericardial catheter, but she remained hypotensive. A bedside echo showed a large pericardial effusion (Figure 3) with near collapse of the ventricles signifying pericardial tamponade.

We suspected a cardiac perforation, and she was immediately taken to the operating room (OR) for mediastinal exploration and control of bleeding. In the OR, it was revealed that she had transection of the distal portion of the LIMA, which was causing the bleeding. The pericardial sac was also opened revealing a large amount of blood that was drained. The accompanying cardiac structures such as the right and left ventricles, the left and right atriums, the pulmonary veins, and ascending aorta were inspected and no obvious damage was noted. The LIMA was clipped and ligated at several points to control the bleeding, following which her blood pressure improved and we were able to wean her off norepinephrine. The patient had an uneventful rest of the hospital stay and was soon discharged to a skilled nursing facility.

## Discussion

Pericardiocentesis is an invasive procedure often used in the management of large symptomatic pericardial effusion and cardiac tamponade. Though it was first described in 1653 by Riolanus, the technique and application of pericardiocentesis have grown over the years [1]. Ultrasound-guided pericardiocentesis has become the gold standard of performing this procedure and has decreased the rate of major complications to around 1.5% [4].

Pericardiocentesis is often lifesaving and can be performed in any clinical setting. In a non-emergent setting, it is preferable to do it in the catheterization lab under fluoroscopy and echo guidance to minimize the risks of complications. There are three main approaches used for pericardiocentesis: (1) subxiphoid (subcostal), (2) apical, and (3) parasternal approach (intercostal). The subxiphoid approach is the most commonly used technique and is also the safest approach in the absence of imaging guidance [5-7]. It involves introducing the needle between the xiphisternum and the left costal margin at an angle of 30-45 degrees, directed towards the left shoulder. The apical approach involves introducing the needle 1 cm lateral to the apical impulse angled towards the right shoulder. Because of the high risk of ventricular puncture and ventricular fibrillation, this approach is not recommended in an emergency [8]. The parasternal approach involves introducing the needle through the left fifth intercostal space (superior to the rib to avoid damage to intercostal vessels and nerve) and 1-2 cm lateral to the sternum. The risk of damaging the LIMA is high with this procedure as the artery runs 1 cm parallel to the sternum but avoids damage to the diaphragm and phrenic nerve [9]. 

Since the advent of ultrasound-directed pericardiocentesis, echo has been widely used to determine the site of entry. This usually depends on the area on the chest wall that is closest to the maximal fluid collection and a trajectory that avoids nearby vital structures [10]. Fluoroscopy-guided pericardiocentesis is also an alternative approach that can be used, especially in stable patients and in the management of post-surgical pericardial effusions. It is considered a very safe procedure, is usually preceded by echocardiographic visualization, and is performed via the subxiphoid approach. In a study by Ibrahim et al, the incidence of complications seen with fluoroscopic guidance was close to zero [11]. However, this procedure is performed only in the catheterization lab and involves exposing the patient and the physician to significant radiation.

Several complications have been reported following pericardiocentesis such as pneumothorax, hemothorax, injury to the heart and coronary vessels, injury to internal thoracic vessels, injury to lung parenchyma or phrenic nerve, injury to abdominal viscera, and ventricular fibrillation [2-4,12]. However, injury to the internal mammary artery as a result of pericardiocentesis has been very rarely noted.

The internal mammary artery arises from the subclavian artery and descends anteromedially into the thorax. It runs along the inner wall of the rib cage and is located around 2-3 cm lateral to the sternum on either side. In most cases, it ends at the seventh intercostal space as a bifurcation into the musculophrenic and superior epigastric arteries. The musculophrenic arteries run lateral and downwards along the costal margin and supply the diaphragm and pericardium. The superior epigastric arteries run inferiorly along the abdominal wall to join with the inferior epigastric artery at the umbilicus. Though the internal mammary artery can be damaged from sharp chest-wall trauma or blunt chest injury, it is rarely reported to be affected during pericardiocentesis [13]. Mehra et al. reported a case of LIMA pseudoaneurysm following pericardiocentesis via the apical approach [14]. 

Our patient underwent pericardiocentesis under fluoroscopy guidance via the subxiphoid approach. It is possible that the LIMA might have been transected in the path of the needle. The visualization of a transected LIMA by the surgeon in the OR, the blood noted in the rectus sheath, recurrence of pericardial tamponade, and the patient’s clinical and hemodynamic improvement following ligation of the bleeding vessel confirm that it was bleeding from the LIMA that had led to the acute decompensation of our patient. To the best of our knowledge, this is the first reported case of injury to the LIMA from pericardiocentesis done via the subxiphoid approach. 

Limitations

We acknowledge the lack of intraoperative images showing the transected LIMA, but through the detailed intraoperative report by the cardiothoracic surgeon and our discussion with him, we have been able to confirm this. We have also not been able to completely explain why the patient did not have any hemodynamic compromise or increased bleeding until a few hours after the procedure. We believe that since the effusion was completely drained, it might have been building up in the interim leading to tamponade and the drain position might not have been appropriate in the beginning to drain it.

## Conclusions

Damage to the LIMA is a possible complication of pericardiocentesis, occurring not just with the parasternal approach, but also with the subxiphoid approach. Through our case report, we urge other physicians to be aware of this potentially catastrophic complication during pericardiocentesis and to continue to report similar incidents. We also recommend close observation of patients following pericardiocentesis to be able to diagnose and manage such complications early.
